# Oral health and salivary function in ulcerative colitis patients

**DOI:** 10.1177/2050640620957138

**Published:** 2020-09-02

**Authors:** Ariana Goldinova, Christopher XW Tan, Gerd Bouma, Marjolijn Duijvestein, Henk S Brand, Nanne K de Boer

**Affiliations:** 1 Department of Gastroenterology and Hepatology, Amsterdam UMC, Vrije Universiteit Amsterdam, AGEM institute, Amsterdam, The Netherlands; 2 Departments of Oral and Maxillofacial Surgery/Oral Pathology, Amsterdam UMC/Academic Centre for Dentistry Amsterdam, Amsterdam, The Netherlands; 3 Department of Oral Biochemistry, Academic Centre for Dentistry Amsterdam, Amsterdam, The Netherlands

**Keywords:** Ulcerative colitis, inflammatory bowel disease, oral, saliva, dental, xerostomia, MUC5B

## Abstract

**Background:**

Although ulcerative colitis primarily involves the colon, extra-intestinal manifestations are common and oral and dental complaints are no exception.

**Objective:**

This study aims at evaluating oral and dental health problems and salivary function and composition in ulcerative colitis patients and its correlation with disease activity.

**Methods:**

Xerostomia Inventory score, (unstimulated/stimulated) salivary flow rates, salivary amylase and mucin/ Mucin 5B levels, self-reported oral and dental complaints, the oral health related quality of life, Simple Clinical Colitis Activity Index and inflammatory bowel disease-specific health related quality of life were determined.

**Results:**

The cohort consisted of 51 ulcerative colitis patients. Hyposalivation was experienced by 16% of patients under resting conditions and 24% under chewing-stimulated conditions. Xerostomia was not correlated with salivary flow rates. Disease activity did not influence salivary amylase and Mucin 5B concentrations. The Xerostomia Inventory score was correlated with the Simple Clinical Colitis Activity Index (*p* = 0.042) and inflammatory bowel disease-specific health related quality of life (*p* = 0.001). Most reported oral health problems were halitosis (29%) and aphthae (28%). Frequently reported dental problems were cavities (35%) and gum problems (31%). Patients with active disease experienced significantly more oral and dental complaints. The number of oral problems was positively correlated with the Simple Clinical Colitis Activity Index (*p* = 0.045) and negatively correlated with the inflammatory bowel disease-specific health related quality of life (*p* = 0.005).

**Conclusion:**

The subjective feeling of a dry mouth (xerostomia) is related to disease activity and disease activity-associated quality of life in ulcerative colitis patients, whereas the objective saliva secretion rate is not. Oral and dental health problems are frequently observed in patients with ulcerative colitis, especially during active disease.

## Key Summary

### Summarise the established knowledge on this subject

- Extra-intestinal manifestations are frequently encountered in patients with ulcerative colitis. The most common sites include the skin, eyes, joints and liver. The oral cavity is also considered as an extra- intestinal site but far less studied.
- Ulcerative colitis is associated with specific and nonspecific oral signs and symptoms as halitosis, dry mouth, aphthous ulcers, pyostomatitis vegetans and lichen planus.
- Saliva is important for the maintenance of oral and general homeostasis. It has a crucial function in the digestion, hydration of the oral mucosa and protection of the teeth.

### What are the significant and/or new findings of this study?

- Patients with active ulcerative colitis experienced significantly more oral and dental complaints with a negative impact on the quality of life.
- Xerostomia, the subjective feeling of a dry mouth, was not correlated with salivary flow rates.
- Disease activity did not influence salivary amylase and MUC5B concentrations.

## Introduction

Ulcerative colitis (UC) is a chronic inflammatory disorder of the gastrointestinal tract, which, together with Crohn’s disease (CD), belongs to the family of inflammatory bowel diseases (IBD). The aetiology is considered to be a combination of genetic predisposition, environmental factors and a dysbiotic microbiota with an excessive host response.

Although the clinical presentation of patients with UC primarily involves intestinal symptoms such as abdominal pain, diarrhoea and rectal bleeding, extra-intestinal manifestations (EIM) are frequently encountered. The most common EIM are located in the skin, eyes, joints and liver. The oral cavity is also considered an extra-intestinal site of involvement in UC.^1–3^

In the literature, limited studies have reported inconclusive results on the prevalence of oral and dental complaints in patients with UC.^4,5^ UC is associated with specific and nonspecific oral signs and symptoms such as halitosis, dry mouth, aphthous ulcers, pyostomatitis vegetans and lichen planus.^6^ Some oral manifestations seem specifically correlated with disease activity.^6^

Saliva is important for the maintenance of oral and general homeostasis. It has a crucial function in digestion, hydration of the oral mucosa and protection of the teeth. Caries, periodontal disease and other oral inflammation may be caused by a lack of antimicrobial peptides present in saliva, sometimes due to hyposalivation.^7–9^

Salivary dysfunction might result in a reduced salivary flow rate (SFR, hyposalivation), the subjective feeling of a dry mouth (xerostomia) and/or an altered biochemical composition of saliva. Although xerostomia is frequently a manifestation of reduced salivary flow, it can also be a symptom on its own. Hyposalivation can be a disabling condition, as it may cause problems with eating, speaking and sleeping and can, therefore, have a significant negative effect on a patient’s quality of life.^9^

Saliva is mainly secreted by three pairs of salivary glands: parotid, submandibular and sublingual. Mucins, mainly secreted in saliva by the submandibular and sublingual glands, are large glycoproteins that provide lubrication and microbial protection to the oral cavity. The visco-elasticity of saliva is mainly dependent on the concentration of Mucin 5B (MUC5B).^10^ MUC5B retains water in the oral mucosa and therefore has an important influence on the perception of a dry mouth.^11^ Amylase in saliva is particularly secreted by the parotid glands and splits high molecular-weight carbohydrates into lower molecular weight sugars.^12^ Amylase also has an important role in maintaining mucosal immunity.^12,13^

Previous studies observed higher salivary concentrations of MUC5B and amylase during inflammation, possibly to protect the oral cavity by decreasing the flow rate of the salivary glands.^14^ A comparable study in CD patients found a significantly higher concentration of MUC5B during active intestinal disease. This indicates a difference in salivary composition in patients with active disease.^15^

The aim of this study was to evaluate oral health problems and salivary function and composition in UC patients and its correlation with disease activity. Secondary, we wanted to evaluate the correlation between quality of life and oral health problems in UC patients with active and inactive disease activity.

## Material and methods

### Study design and participants

This was a cross-sectional cohort study in a tertiary referral center for IBD. The study population consisted of consecutive patients with UC who visited the outpatient IBD clinic or day care unit of the Amsterdam UMC (location VUmc). Due to the absence of literature data, no power analysis could be performed. It was estimated that at least 50 subjects would be sufficient.

Inclusion criteria were a confirmed diagnosis of UC and an age of 18 years or older. Exclusion criteria were inability to read or speak Dutch and pregnancy. Subjects were asked to stay an additional 30 minutes to participate in salivary flow test and fill in five (inter)nationally validated questionnaires about their oral health, intestinal disease activity and oral health and IBD-specific health-related quality of life.

### Healthy controls

A formal control group was not included in the study. However, data from oral and dental health were obtained from a study by Kalsbeek in 2003.^16^ This study investigated in detail the oral and dental health in a large cohort of healthy Dutch individuals (*n* = 1407) after a change in the insurance system after 1995 (so-called Nederlandse Organisatie voor Toegepast-Natuurwetenschappelijk Onderzoek (TNO) cohort).

### Whole saliva secretion rates

Saliva was collected from all participating subjects. Unstimulated whole saliva (UWS) and chewing-stimulated whole saliva (CWS) were collected with the spitting method.^17^ The subjects were instructed to refrain from eating, smoking and drinking during at least 1 hour prior to the collection of saliva; tap water was allowed. Before the collection of UWS, subjects were informed to swallow saliva still present in their mouth and then to drool produced saliva for 5 minutes in a pre-weighed container. The subjects were not allowed to talk, swallow or move the tongue during these 5 minutes. To obtain CWS the procedure was repeated while the patients were asked to chew on a piece of tasteless Parafilm (5 × 5 cm; 0.03 g). By re-weighing the containers, we calculated the SFR, assuming 1 gram is 1 millilitre.^9^ Cut-off values for hyposalivation were 0.1 mL/minute for resting whole saliva and 0.7 ml/minute for CWS.^18^

### Questionnaires

Five different questionnaires were used in this study. To quantify the xerostomia experienced by patients the Xerostomia Inventory (XI) was used. This is a validated and frequently used questionnaire that consists of 11 items, each on a five-point Likert scale.^19^ The presence of oral- and dental complaints was determined by an oral health questionnaire (OHQ). The OHQ contains questions about different oral and dental complaints experienced by patients during the last 12 months. The questions need to be answered with yes or no. The Oral Health Impact Profile (OHIP-14) has been developed to assess the quality of life in relation to oral health.^20^ It consists of 14 items, each on a five-point Likert scale from 0 (never) to 4 (very often) according to the frequency of the impact on the quality of life. The Simple Clinical Colitis Activity Index (SCCAI) was used to measure intestinal disease activity. A disease activity score of five or higher indicates active disease and a disease activity score under five means inactive disease.^21^ To measure intestinal disease activity and its influence on the patient’s quality of life the shortened and validated IBD Questionnaire (IBDQ-9) was used.^22^ Responses to each item of the IBDQ-9 were scored on a seven-point Likert scale, where a score of one indicates the worst and a score of seven the best possible condition. The scores of the individual items are summed resulting in a total IBDQ-9 score ranging from nine to 63 with higher scores reflecting a better wellbeing of patients.

### Amylase and MUC5B activity in saliva

The collected UWS and the CWS saliva samples were vortexed and centrifugated. The samples were then 1:1 diluted with 500 mM NaCl and stored at –70°C until biochemical analysis. To determine the amylase activity, EnzChek® Ultra Amylase Assay Kit was used according to the manufacturer’s instructions. The saliva samples were used in a dilution of 1:100 amylase CNPG3 buffer. The reaction was measured every minute for 20 minutes and enzyme activity per minute was determined using a calibration curve. MUC5B concentration was determined by enzyme-linked immunosorbent assay, using pooled saliva of healthy persons as standard reference.^23^ For both biochemical analyses, polystyrene microtiter 96 wells plates were used (Greiner Bio-One microlon nr. 655151 and 655092), using a Multiscan FC microplate photometer (Thermo Scientific). The amylase and MUC5B activity are expressed in unit/millilitres (U/mL).

### Statistical analyses

SPSS-Statistics version 25 was used for statistical analyses. The significance level was set at *p*<0.05. The primary outcomes were the XI-score, UWS and CWS. To analyse whether data were normally distributed, Kolmogorov-Smirnov and Shapiro-Wilks tests were used. The Spearman correlation test was used to analyse the correlation between the primary outcomes and IBDQ-9, SCCAI and number of oral and dental problems. Based on the SCCAI score, the study population was dichotomized into a group with active and a group with inactive disease (SCCAI score of ≥5 and <5, respectively). Chi-square tests were used to compare individual oral and dental complaints in active and inactive disease.

## Ethical considerations

The study design was reviewed and approved prior to the start of the study by the Medical Ethic Committee of the Amsterdam UMC, location VUmc (number 2018.624 and date November 2018). All subjects gave informed written consent preceding their participation in the study.

## Results

### Cohort characterization

Demographic and disease specific characteristics of the cohort of UC patients are depicted in Table 1. A total of 51 patients were included in the study. One patient was not able to perform the CWS and therefore registered as missing for this part of the study.

**Table 1.** Demographic data of included ulcerative colitis patients.

Characteristics	Total *n* = 51	Active (SCCAI≥ 5)*n* = 14	Inactive (SCCAI<5)*n* = 37	*p*
Age	43.8 ± 16.3	49.64 ± 18.2	40.92 ± 15.4	0.094
Participants				
- Female	27 (52.9%)	8 (57.1%)	19 (51.4%)	0.762
- Male	24 (47.1%)	6 (42.9%)	18 (48.6%)	
Smoking				0.533^a^
- Yes	6 (11.8%)	1 (7.1%)	5 (13.5%)	
Medication (three missing data)	*n* = 48	*n* = 12	*n* = 36	0.852^a^
- None	5 (10.4%)	2 (16.7%)	3 (8.3%)	
- Aminosalicylates	17 (35.4%)	4 (33.3%)	13 (36.1%)	
- Corticosteroids	0	0	0	
- Immunosuppressants	9 (18.8%)	1 (8.3%)	8 (22.2%)	
- Anti-TNF treatment	16 (33.3%)	5 (41.7%)	11 (30.6%)	
- Combination Aminosalicylates and Anti-TNF	1 (2.1%)	0 (0%)	1 (2.8%)	
Disease extent (Montreal classification) (four missing data)	*n* = 47	*n* = 12	*n* = 35	0.494^a^
E1 Ulcerative proctitis	9 (19.1%)	2 (16.7%)	7 (20.0%)	
E2 Left-sided colitis	19 (40.4%)	4 (33.3%)	15 (42.9%)	
E3 Pancolitis	19 (40.4%)	6 (50.0%)	13 (37.1%)	

Data are presented as mean ± standard deviation or number and percentage (%). Chi-square tests for categorical variables and students *t* test for continuous variables.

a Mann-Whitney U test.

UC: ulcerative colitis; SCCAI: Simple Clinical Colitis Activity Index.

The average age of the cohort was 43.4 ± 16.5 years, the minimum age was 18 years old and the maximum age was 77 years old. In total, 27 patients were female (52.9%) and 24 (47.1%) were male. Six of the subjects were smokers (12%). Based on the SCCAI score, 14 of the included 51 patients had active disease, whereas 37 patients were in remission. Of the cohort, 60% had a history of corticosteroid treatment and 40% had not.

### Disease activity associated with oral and dental complaints

In Table 2 the SCCAI was used to compare the frequency of oral and dental problems of the patients with active and inactive UC. Patients with active disease reported significantly more complaints of bad taste, reduced taste, halitosis and missing or broken teeth compared to patients in remission.

**Table 2.** Oral and dental complaints stratified according to disease activity scores.

Oral complaints	Total *n* = 51	Active (SCCAI≥5) *n* = 14	Inactive (SCCAI<5)*n* = 37	*p*
Problems with eating or drinking	6 (11.8%)	2 (14.3%)	4 (10.8%)	0.731
Temporomandibular joint complaints	9 (17.6%)	3 (21,4%)	6 (16.2%)	0.663
Aphthae	14 (27.5%)	3 (21.4%)	11 (29.7%)	0.553
Red or white discolouring of mouth mucosa	4 (7.8%)	2 (14.3%)	2 (5.4%)	0.292
Angular cheilitis	11 (21.6%)	3 (21.4%)	8 (21.6%)	0.988
Irritated mouth mucosa	7 (13.7%)	2 (14.3%)	5 (13.5%)	0.943
Bad taste	11.0 (21.6%)	7 (50.0%)	4 (10.8%)	**0.002** ^a^
Reduced taste	9 (17.6%)	5 (35.7%)	4 (10.8%)	**0.037** ^a^
Halitosis	15 (29.4%)	7 (50.0%)	8 (21.6%)	**0.047** ^a^
Difficulty speaking	5 (9.8%)	3 (21.4%)	2 (5.4%)	0.086
Oral fungal infection/thrush	2 (3.9%)	1 (7.1%)	1 (2.7%)	0.466
Pain	10 (19.6%)	4 (28.6%)	6 (16.2%)	0.321
Burning tongue	5 (9.8%)	2 (14.3%)	3 (8.1%)	0.508
Dental complaints				
Cavities	18 (35.3%)	5 (35.7%)	13 (35.1%)	0.969
Gum problems	16 (31.4%)	3 (21.4%)	13 (35.1%)	0.346
Loosening, missing or broken teeth	8 (15.7%)	6 (42.9%)	2 (5.4%)	**0.001** ^a^
Teeth malposition	6 (11.8%)	3 (21.4%)	3 (8.1%)	0.188
Sharp edges of teeth	7 (13.7%)	3 (21.4%)	4 (10.8%)	0.325
Sensitive root surfaces	13 (25.5%)	3 (21.4%)	10 (27.0%)	0.682

a Statistically significant, *p* < 0.05.

UC: ulcerative colitis, SCCAI: simple clinical colitis activity index.

### Oral and dental complaints in patients versus healthy subjects

The oral and dental complaints reported by the UC patients are presented in Table 2. Commonly reported oral health problems were aphthae, angular cheilitis, bad taste and halitosis. Frequently witnessed dental problems were cavities, gum problems and sensitive root surfaces. Compared with a control group of 1407 healthy Dutch inhabitants (Table 3) differences in prevalence were found for aphthae, temporomandibular joint complaints, bad taste, halitosis and cavities.

**Table 3.** Oral and dental complaints compared to healthy controls.

	UC patients	Controls
Oral	*n* = 51	*n* = 1407
Problems with eating or drinking	11.8%	17%
Temporomandibular joint complaints	17.6%	7%
Oral blisters or ulcers	27.5%	9%
Red or white discolouring of mouth mucosa	7.8%	–
Angular cheilitis	21.6%	–
Irritated mouth mucosa	13.7%	–
Bad taste	21.6%	5%
Reduced taste	17.6%	–
Halitosis	29.4%	10%
Difficulty speaking	9.8%	–
Oral-fungal infection/thrush	3.9%	–
Pain	19.6%	13%
Burning tongue	9.8%	–
Dental		
Cavities	35.3%	25%
Gum problems	31.4%	24%
Loosening, missing or broken teeth	15.7%	22%
Teeth malposition	11.8%	14%
Sharp edges of teeth	13.7%	13%
Sensitive root surfaces	25.5%	–

UC: ulcerative colitis.

### Saliva and hyposalivation

The XI-score was normally distributed with a mean XI-score of 23, ranging from 11 to 44.

The UWS flow rate was not normally distributed, with a median UWS of 0.26 mL/minute ranging from 0.04 to 1.40 mL/minute. Hyposalivation under resting conditions was observed in eight of the 51 patients (16%). The CWS flow rate was normally distributed. The mean CWS rate was 1.31 mL/minute ranging from 0.06 to 3.42 mL/minute. Hyposalivation under chewing-stimulated conditions was observed in 12 of 50 patients (24%). No statistically significant correlations were detected between XI and UWS (*r* = −0.009, *p* = 0.953) and between XI and CWS (*r* = −0.186, *p* = 0.196), respectively.

### Correlations of intestinal disease activity with salivary function

The correlations between SCCAI and XI, UWS, CWS, salivary MUC5B and salivary amylase were analysed. A positive correlation was observed between SCCAI and XI-score (*r* = 0.285, *p* = 0.042), indicating that subjects with active disease had a more severe feeling of a dry mouth. SCCAI did not correlate with the unstimulated and stimulated saliva secretion rates (UWS *r* = 0.139, *p* = 0.332 and CWS *r* = −0.17, *p* = 0.237). The SCCAI also did not correlate with the salivary amylase activity, the MUC5B concentrations or the ratios of amylase:MUC5B in both stimulated and unstimulated saliva, respectively.

A significant correlation was found between the IBDQ-9 score and the XI-score (*r* = −0.527, *p* = 0.001). No correlation was observed between IBDQ-9 scores and the salivary amylase activity, the MUC5B concentrations or the ratios of amylase:MUC5B in both stimulated and unstimulated saliva.

### Correlations of disease duration and salivary function

The disease duration in this cohort ranged from 0–39 years, with a mean duration of 14 years.

No statistically significant correlation was found between disease duration and the XI-score (*r* = −0.099, *p* = 0.523). Also, no statistically significant correlations were found between disease duration and UWS (*r* = −0.76, *p* = 0.622) and between disease duration and CWS (*r* = 0.103, *p* = 0.507), respectively.

### Amylase and MUC5B

The median amylase activity for UWS was 87.3 U/mL (range 0.7–836.3). The median amylase activity for CWS was 99.3 unit/mL (range 0.2–444.5). The median of the MUC5B concentration for UWS was 0.7 unit/mL (range 0–4.4) and for CWS 0.3 unit/mL (range 0–2.3). The median of the amylase:MUC5B ratio was 52.4 (range 0–2566.9) for UWS and 182.9 (range 8.3–14,821) for CWS. The XI score was not significantly correlated to the salivary amylase activity, MUC5B concentration or the amylase:MUC5B ratio.

### Oral health-related quality of life in UC patients with active and inactive disease

A near significant correlation of the OHIP-14 scores with SCCAI and IBDQ-9 was observed (*r* = 0.280, *p* = 0.051 and *r* = −0.274, *p* = 0.057, respectively). A positive relation was found between the number of oral and dental complaints of patients and the IBDQ-9 score (*r* = 0.393, *p* = 0.005). A significant correlation was also observed between the number of oral problems and SCCAI (*r* = −0.285, *p* = 0.045).

## Discussion

As far as we know, this is the first study that explored oral health problems and salivary function in correlation with intestinal disease activity in patients with UC. Active disease was associated with more xerostomia and a diminished quality of life, but not with objective oral dryness. No correlation was observed between the volume of secreted unstimulated and stimulated whole saliva with xerostomia. Moreover, we observed that oral and dental problems are frequently present in patients with UC, especially during active disease.

We found that the median XI-score of our cohort was 22. In a previously reported study, the median score of the healthy controls was almost 17.^24,25^ In the UWS sample group, 15.7% experienced hyposalivation, which is more than in a general healthy cohort (10%) and in the CWS group 24% experienced hyposalivation, compared with 0–5% in a general population. We therefore assume that UC patients experience more severe xerostomia. A possible explanation for the xerostomia without objective oral dryness might be due to alterations during active disease in the visco-elastotic properties of the patients’ whole saliva, possibly by diminished minor salivary gland secretion, failing to lubricate the mouth properly.^24^ Contrary to our expectations, no relation was found between the severity of xerostomia and salivary composition. Also, no correlation was found between intestinal disease activity and MUC5B or amylase, respectively.

A higher percentage of several oral complaints was observed when we compared our UC cohort with the cohort of healthy controls. Patients with active disease reported bad taste, reduced taste, halitosis and missing or broken teeth more often when compared to patients with UC in remission. These findings are roughly in line with current literature. In a comparative Iranian cohort of 50 patients, a significant statistical relationship was found among tongue coating, halitosis and oral ulceration in patients with severe UC compared to the control group.^4^

Recently, a similarly designed study examined xerostomia, salivary function and oral health problems among a cohort of 53 Dutch patients with CD.^15^ In patients with CD no significant correlation between disease activity and xerostomia was observed. In contrast, in CD patients salivary MUC5B correlated with disease activity whereas in UC patients no correlation was found. These opposite observations might be related to the unique rheological properties of MUC5B. It contributes to the formation of the thin salivary film on the oral mucosa, which plays an important role in dry mouth perception.^26^ Patients treated with radiotherapy for head-and-neck cancer with higher salivary MUC5B levels suffer less from xerostomia than patients with low levels of MUC5B in saliva.^27^ Similarly, in CD patients the disease-associated increase in salivary MUC5B concentration could counteract the expected increase in the severity of xerostomia.

We also found that patients in the UC group experienced more frequent oral complaints compared to previously described CD patients.^15^ This is in line with a previous review that concluded oral involvement is more pronounced in CD than UC^28^ and that active inflammation has a negative influence.^29^ A systemic review of nine cross-sectional studies including 1297 IBD patients also concluded that UC patients had worse oral health compared to CD patients.^30^ The difference in oral health might be related to differences in immunological responses in both diseases. CD is considered to be a Th1 disease whereas UC has more characteristics of a Th2-type disease.^31^

As mentioned in the introduction, the aetiology of UC is considered to be a combination of genetic predisposition, environmental factors and a dysbiotic microbiota with an excessive host response. Proteobacteria are one of the most common phyla in the human gut microbiota and are found at different body sites, such as skin, tongue, vaginal tract and in the oral cavity.^32^ Proteobacteria are often found to be increased in UC, indicating these microorganisms may carry proinflammatory characteristics.^33^ There might be a relationship between oral health problems, disease activity of UC and proteobacteria.

Recently, Nanki and co-workers^34^ demonstrated by exon sequencing that somatic PIGR mutations in UC result in concomitant depletion of secretory IgA in the intestinal epithelium. Immunoglobulin IgA contributes to the maintenance of the mucosal homeostasis in the colon^35^ and regulates the composition and function of gut microbiota.^36^

Only a limited number of studies have investigated salivary IgA in UC and their results are inconclusive. Two studies reported that salivary IgA concentration did not differ between UC and healthy subjects.^37,38^ However, another study reported a significant negative correlation between disease activity and the concentration of IgA in whole saliva.^39^ Therefore further studies on the salivary IgA concentration and its possible role in oral health problems in UC seem warranted.

The results of our study should be interpreted with some caution as we included a relatively small number of patients. The oral cavity was not inspected by a professional, instead we used questionnaires to gain an insight into the oral and dental health of UC patients. This could be considered a limitation, because patients might not be aware of all their oral and dental problems, therefore we could have missed some problems or overestimated others.

Also, we could not correct for potential effect modification and confounding, because of the small sample size. Comorbidities, medication, age, site of involvement, gender and nutritional state were not taken into account.

Another limitation of the present study is that it did not include a cohort of age- and gender-matched healthy subjects as a control group, but historical data from a large Dutch cohort from the general population.^16^ However, this historical cohort appears to be quite comparable with regard to mean age (40 years) and the gender distribution (58% female, 42% male) with the cohort of UC patients in the present study (mean age 43.8 years, 53% female). Also, this study took place in a tertiary referral center, which may influence the complexity of the disease and the type of medication used by the included patients.

Our study underlines that oral health problems are common in patients with UC, especially during active disease, therefore we plea for a better communication and collaboration between gastroenterologists and dentists to strive for optimal care for UC patients. Moreover, understanding of the oral presentation of UC may improve early diagnosis and may even prevent pain and discomfort of the oral cavity and teeth. It is essential to inform the patient about the possible consequences of UC for the oral cavity.

## Conclusion

The subjective feeling of a dry mouth (xerostomia) is related to disease activity and disease activity-associated quality of life in UC patients, whereas the objective saliva secretion rate is not. Oral and dental health problems are frequently observed in patients with UC, especially during active disease.
